# CyDye Immunoblotting for Proteomics: Co-detection of specific immunoreactive and total protein profiles

**DOI:** 10.1002/pmic.200600139

**Published:** 2006-12

**Authors:** Pamela M Donoghue, Ciara A McManus, Niaobh M O'Donoghue, Stephen R Pennington, Michael J Dunn

**Affiliations:** Proteome Research Centre, UCD Conway Institute of Biomolecular and Biomedical ResearchDublin, Ireland

**Keywords:** Co-detection, CyDye immunoblotting, ECL-Plex, Heart, Western blotting

## Abstract

The development of ECL-Plex CyDye-conjugated secondary antibodies allows the advancement of conventional Western blotting, opening up possibilities for highly sensitive and quantitative protein confirmation and identification. We report a novel proteomic method to simultaneously visualise the total protein profile as well as the specific immunodetection of an individual protein species by combining cyanine CyDye pre-labelled proteins and antibody immunoblotting. This technique proposes to revolutionise both 2-D immunoprobing and protein confirmation following MS analysis.

Western immunoblotting facilitates the interaction of immobilised proteins with specific polyclonal or monoclonal antibodies, identifying the corresponding immunoreactive protein. This technique, developed in the 1970s, is still widely used as a rapid and relatively straightforward method of protein identification [1]. Conventional immunodetection, however, does not move easily from 1-D to 2-DE. Two-dimensional gels are able to display simultaneously up to 2000 protein products on a single gel [2]. Immunoblotting for a specific protein species can therefore be extremely difficult, as it is often impossible to say definitively which spot on the 2-D gel correlates with the immunoreactive protein. A number of methods for pre- and post-staining of total protein attempt to address this problem. These include gel-staining techniques such as CBB and silver stain along with fluorescence-based methods such as SYPRO-Ruby and Deep Purple. Pre-staining does present an overall impression of the total protein profile but many of these methods are incompatible with downstream immunoblotting analysis and, therefore, must be removed from the blot membrane prior to antibody detection. Protein stains such as Amido black, Ponceau-S, Fast green FC, Colloidal gold and India ink all successfully stain proteins bound to immunoblot membranes but are again lost during antibody incubation [3].

The method described here, based on the use of the recently developed ECL Plex-CyDye immunoblot detection system (GE Healthcare, Uppsala, Sweden), allows the simultaneous immunodetection of an individual protein species in conjunction with visualisation of the total protein profile. Protein samples are initially pre-labelled with the charge-matched “minimal labelling” cyanine CyDye reagents (GE Healthcare), Cy3 and Cy5, and then separated by 1-D or 2-DE. Following electrophoresis, the proteins are transferred by electroblotting onto membranes and probed with a specific primary antibody of choice. Subsequent incubation of the membrane with wavelength-specific ECL Plex fluor-labelled species-specific secondary antibodies (GE Healthcare) enables the visualisation of both the total protein expression profile and the specific immunoreactive pro-tein(s). This technique is not only highly sensitive, as a consequence of the use of CyDye labelling, but also reduces the number of gel replicates and amount of sample needed. Protein spots of interest can then be easily correlated with other analytical or micro-preparative 2-D gels for further differential expression analysis and/or protein identification by MS.

Human left ventricular heart tissue (0.2 g) was ground to a fine powder with the aid of liquid nitrogen and a pestle and mortar. Ground tissue (0.25 g) was immediately added to DIGE compatible lysis buffer (9.5 M urea w/v, 2% CHAPS w/v, and 20 mM Tris w/v pH 8.0–8.5) and incubated at room temperature for 1 h. Protein samples were sonicated on ice for 3 × 10-s bursts. Following centrifugation at 14000 × *g*_av_ for 30 min, protein fractions were collected, aliquoted and stored at −80°C until further use. All preparative steps were performed at 0–4°C in the presence of a protease inhibitor (Roche complete protease inhibitor cocktail tablets) to prevent proteolytic degradation. Protein concentration was determined using the Bradford dye-binding assay [4].

Minimal CyDye labelling (GE Healthcare) was performed at a concentration of 25 μg of protein/200 pmol of CyDye for 1-DE. Due to the separation capabilities of 2-DE a minimum protein loading of 150 μg/1000 pmol of CyDye was required for 2-DE analysis. Protein loadings of 300 and 600 μg were labelled at the same ratio. Labelled Cy 3 and Cy 5 samples were incubated on ice for 30 min in the dark. The labelling reaction was terminated by the addition of 10 mM lysine. After a brief spin, samples were incubated on ice in the dark for 10 min. Labelled protein fractions were utilised immediately for further analysis or otherwise stored at −20°C. Protein samples analysed by 1-D SDS-PAGE were combined with an equal volume of Laemmli buffer (Bio-Rad, Hemel-Hampstead, UK) while 2-DE samples were combined with an equal volume of 2X lysis buffer (9.5 M urea, 2% CHAPS, 2% DTTand 1.6% Pharmalyte pH 3–10) followed by in-gel rehydration overnight prior to IEF.

1-D SDS-PAGE was carried out using the Bio-Rad mini-gel system on 4—20% gradient gels (Pierce, IL, USA) according to Laemmli *et al.* [5]. Pre-labelled protein samples were combined with an equal volume of Laemmli sample buffer (Bio-Rad), and separated at 160 V for 1.5 h using the Bio-Rad mini-Protean III system. For 2-DE, 150, 300 and 600 μg of human heart left ventricle protein samples were diluted in rehydration solution (8 M urea, 0.5% w/v CHAPS, 0.2% w/v DTT and 0.2% w/v Pharmalyte pH 3–10) and applied to 24-cm IPG strips (pH 3–10, non-linear (NL), GE Healthcare). Strips were rehydrated overnight at room temperature using an in-gel rehydration method [6]. The sample was then subjected to IEF at 0.05 mA/IPG strip for 72 000 Vh at 20°C. The strips were equilibrated in 6 M urea, containing 30% v/v glycerol, 2% w/v SDS, 0.05 M Tris-HCl, pH 8.8 and 0.01% w/v Bromophenol blue with the addition of 1% w/v DTT for 15 min. Subsequently, the strips were equilibrated in the same buffer without DTT but with the addition of 4.8% w/v iodoacetamide for 15 min [7]. The second dimension was carried out overnight using a Bio-Rad Protean Plus Dodeca Cell system at 1 W/gel at 15°C and was terminated when the dye front had just migrated off the lower end of the gels. Gels were subsequently scanned using a Typhoon variable mode imager 9400 (GE Healthcare).

SDS-PAGE gels were electrophoretically transferred onto Hybond NC membranes (GE Healthcare) using a Protean blotting system (Bio-Rad) at 100 V for 1 h in transfer buffer (0.2 mM Tris, 2.5 mM glycine and 20% methanol). Immediately after 2-DE, the gels were equilibrated in buffer (20 mM Tris base, 150 mM glycine pH 8.3) with gentle agitation at room temperature for 30 min. It is necessary to separately soak the NC and filter paper in equilibration buffer to avoid the transfer of artefacts to the membrane. The separated proteins were then transferred by semi-dry blotting to Hybond NC membranes for 2 h at 0.8 mA/cm^2^. Membranes were blocked for 2 h at room temperature in PBS containing 5% BSA and then incubated overnight in anti-Hsp 27 mAb (SPA−800, Stressgen) (1:800 dil) in PBST (PBS, 0.1% Tween 20). Membranes were washed twice in PBST and then incubated in either ECL Plex goat-anti-mouse IgG, Cy 3 or goat-anti-rabbit IgG, Cy 5-conjugated secondary antibody (GE Healthcare) (1:2000 dil) for 1.5 h in PBST protected from light. After incubation in secondary antibodies, blots were washed twice in PBST for 10 min each, followed by two washes in PBS while protected from light. All membranes and immunoblots were visualised at each stage using a Typhoon variable mode imager 9400 with a pixel volume at 40 000–60 000 for all scans. It is also important to use lint-free tissue throughout the experimental protocol especially for membrane storage.

To confirm Hsp 27 immunodetection, tryptic peptides were analysed using an Applied Biosystems 4700 Proteomics Analyser MALDI-TOF-TOF mass spectrometer using 10 mg/mL CHCA as the matrix. Mass spectra were acquired in the reflector mode and the peptide sequences were confirmed by MS/MS of selected precursors. The precursor ion masses and the masses of the daughter ions from MS/MS experiments were scanned against the Swiss-Prot database using MASCOT Search Software incorporated in the GPS Explorer™ v3.5 software. The following search parameters were employed. The UniProt (release 6.0) Swiss-Prot non-redundant database and the human taxonomy were used for all searches. Methionine oxidation and cysteine carboxy-amidomethylation were specified as variable modifications and a maximum of one missed cleavage site was allowed. The precursor tolerance was set at 150 ppm and the MS/MS fragment tolerance was set at 0.25 Da.

The work described here attempts to address not only the problems associated with conventional non-quantitative immunoblotting but also those associated with protein spot confirmation following fluorescent gel electrophoresis and its subsequent software analysis. As seen in Fig. 1, human left ventricular heart proteins were initially pre-labelled with both Cy3 and Cy5 CyDyes and separated according to molecular mass by 1-D SDS-PAGE. Separated proteins were then transferred onto NC membranes followed by immunodetection with Hsp 27 mAb. Hsp 27 is a known cardiac heat shock protein and has been investigated extensively in previous work carried out by this research group [8]. Incubation with CyDye-conjugated secondary antibodies allows the visualisation of both total protein expression and specific antibody-CyDye-labelled proteins. As seen in Fig. 1C, the sensitivity of the goat-anti-mouse IgG Cy3 secondary is inferior to its anti-mouse Cy5 counterpart, therefore the Cy3 pre-labelling followed by the anti-mouse Cy5 secondary combination was used in all subsequent analysis.

**Figure 1:**
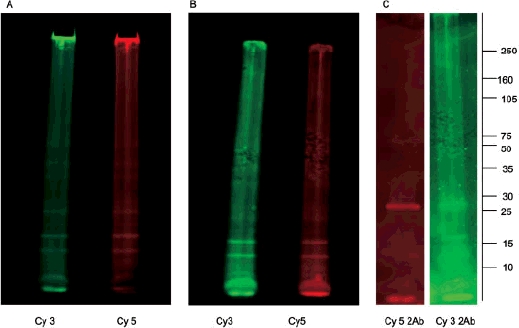
ECL Plex 1-D CyDye blotting. ECL Plex human left ventricular heart fractions pre-labelled with Cy3 and Cy5 CyDyes were electrophoretically separated on a 1-D 4–20% gradient gel (A). Following protein transfer onto NC membranes, (B), blots were probed with Hsp 27 mAb and visualised with Cy5 and Cy3 secondary conjugated antibodies (C). Flour-labelled ECL Plex rainbow molecular mass markers were employed to correctly identify labelled proteins. All gels and membranes were visualised using the Typhoon variable mode imager.

Protein fractions pre-labelled with Cy3 CyDye were subjected to 2-DE at loading concentrations of 150, 300 and 600 μg (Fig. 2), transferred onto NC membranes, incubated with Hsp 27 mAb, and visualised with Cy5-conjugated secondary antibody. As seen in Fig. 2, total protein expression is overlaid with the immunolabelled Hsp 27 protein spots. These images can also be viewed separately by selecting the specific fluor channel for each CyDye (Fig. 2). The multiple immunodetected spots shown in Fig. 2, were manually excised for convenience from a silver-stained micro-preparative 2-D gel. However, it is possible to excise spots from fluorescently stained gels, *e.g.* SYPRO Ruby via a robotics cutter. Spots were subsequently digested with trypsin and identified by MS/ MS as Hsp 27 (Table 1). The multiple spots represent unphosphorylated Hsp 27 and its phosphorylated forms as shown in previous studies [8]. Unlike conventional protein profiling techniques, CyDye immunoblotting can simultaneously reveal both total and specific immunodetected protein components of a complex protein sample. The ability of this technique to visualise the entire expressed proteome combined with immunodetection represents a significant advance for the identification and characterisation of proteins separated by 2-DE.

Multiplex detection of proteins by 1-DE with secondary CyDye- and FITC-conjugated secondary antibodies is addressed by Gingrich *et al.* [9]. Although this method describes the quantitation of protein expression using fluorescent probes it does not incorporate CyDye pre-labelling and post-labelling experiments on either 1-D or 2-D platforms. Leimgruber *et al.* [10] demonstrate a technique incorporating SYPRO-Ruby staining coupled with CyDye labelling and ECL overlaying the total expression profile of treated and non-treated groups. While this technique does demonstrate the ability to view total protein expression before and after protein transfer to a membrane, it does not address CyDye-conjugated antibody immunoblotting or the simultaneous overlay of total protein expression and decorated protein candidates as demonstrated here. The overlay of CyDye-and SYPRO-Ruby-stained images is also questionable, as often many spots do not correctly align between images [11]. Gel and membrane staining methods such as CBB R-250 (500 μg), silver stain (1 ng), Ponceau-S (5 μg) and the reversible membrane stain Memcode (25 ng) [12], do not offer the high level of sensitivity afforded by the combined CyDye and ECL Plex labelling system (1.2 pg). Total protein labelling with India ink (100 ng) or Colloidal gold (4 ng) although highly sensitive, must be removed before immunodetection and therefore limits the comparison between total protein expression and candidate protein species [13].

The step-wise increase in protein loadings used in this study reflects the robustness of this protocol. Experimental replicates (*n* = 3) for each loading subset, 150, 300 and 600 μg, demonstrate the reproducibility of this method. Scanning of 150, 300 and 600 μg gels (*n* = 3) using the Typhoon 9400 showed no interference between increased protein loads and calculated fluorescence as pixel volume was adjusted within a common range (40 000–60 000 pixels) for all scans. As seen in Fig.2, high protein loading does not affect the total protein profile, thus enabling the possible detection of low abundant protein species. The transfer efficiency of CyDye-labelled proteins between gel and membrane was analysed using Progenesis PG240 analysis software (Nonlinear Dynamics). Total spot volume from warped gel and membrane subsets showed a mean transfer efficiency of between 84 ± 9% (150 μg), 74 ± 15% (300 μg) and 86 ± 6(600 μg) (*n* = 3).

**Figure 2:**
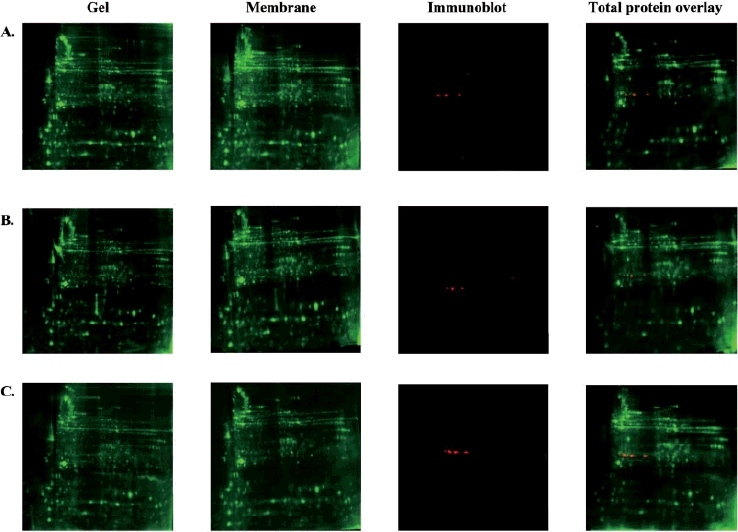
ECL Plex 2-D CyDye blotting. Human left ventricle protein fractions (150 μg (A), 300 μg (B), and 600 μg (C)) were primarily CyDye (Cy3) labelled, focused on pH 3–10 NL IPG strips and subjected to 2-DE using 12% SDS polyacrylamide gels, column 1, (A, B and C). Separated proteins were transferred onto NC membranes, column 2. Primary incubation with Hsp 27 mAb was followed by decoration with CyDye (Cy5)-conjugated secondary antibodies, column 3. Total protein expression combined with specific Hsp 27 protein detection is overlaid and visualised at specific wavelengths for both Cy Dyes, column 4.

**Table 1:** Human left ventricle proteins identified by MALDI-TOF/TOF. The multiple immunodetected spots shown in Fig. 2, column 3, were excised from a silver-stained micro-preparative 2-D gel and identified by MS/MS as Hsp 27 and its isoforms

Spot	Protein identity	Accession no.	MASCOT score	Coverage %	MS/MS peptide no.	Sequence	Charge state
1	Heat shock 27-kDa protein	P04792	75	36	2	LFDQAFGLPR	1
						LATQSNEITIPVTFESR	1
2	Heat shock protein beta 1	P04792	177	66	2	LFDQAFGLPR	1
						LATQSNEITIPVTFESR	1
3	Heat shock protein beta 1	P04792	166	61	3	LFDQAFGLPR	1
						QLSSGVSEIR	1
						LATQSNEITIPVTFESR	1

CyDye immunoblotting also overcomes many of the problems associated with conventional DIGE fluorescent labelling. Labelling of protein mixtures with wavelength-specific CyDyes results in a small but significant shift in pI when visualised on a 2-D gel [10]. This problem is compounded by fluorescent-dependent software analysis packages such as DyCyder and Progenesis. With over 2000 protein spot species on a single 2-D gel, significant spot comparison between DIGE and non-DIGE 2-D gels can prove extremely difficult and can inhibit protein identification in downstream MS analysis. The combination of DIGE and ECL Plex labelling allows the simultaneous comparison of total protein expression with the specific immunodetected protein of interest on the same membrane. The immunodetection of multiple protein targets is also possible with the ECL Plex system as secondary antibodies labelled with different CyDyes can be detected at specific wavelengths, suggesting an added quantitative capability to this approach (http://www5.amershambiosciences.com/applic/28401539.pdf.).

In conclusion, we have demonstrated an advanced CyDye immunoblotting methodology that allows the co-detection of specific immunolabelled protein(s) in conjunction with total proteome expression. This technique provides a simple, rapid and reproducible method for protein confirmation following expression software analysis and/or MS identification.
